# Aspirin in combination with clopidogrel in the treatment of acute myocardial infarction patients undergoing percutaneous coronary intervention

**DOI:** 10.12669/pjms.35.2.87

**Published:** 2019

**Authors:** Xiaoyan Zhang, Lizhen Qi, Yongxuan Liu

**Affiliations:** 1 *Xiaoyan Zhang, Interventional Operating Theater, Binzhou People’s Hospital, Shandong, 256610, China*; 2 *Lizhen Qi, Nuclear Medicine, Binzhou People’s Hospital, Shandong, 256610, China*; 3 *Yongxuan Liu, Department of Cardiology, Binzhou People’s Hospital, Shandong, 256610, China*

**Keywords:** Acute myocardial infarction, PCI, Aspirin, Clopidogrel

## Abstract

**Objective:**

To investigate the clinical effect of aspirin combined with clopidogrel on acute myocardial infarction after percutaneous coronary intervention (PCI).

**Methods:**

One hundred thirty two patients with acute myocardial infarction who were admitted to the hospital between December 2016 and December 2017 were divided into a control group and an observation group according to random number table, 66 each group. Both groups were given emergency PCI and symptomatic treatment. The control group was given aspirin on the basis of conventional treatment before and after operation, while the observation group was given clopidogrel treatment on the basis of the treatment the same as the control group. The treatment lasted for 4 months. The clinical efficacy of the two groups was analyzed, and the cardiac function indicator, coagulation indicator and occurrence of adverse reactions were compared before and after treatment.

**Results:**

There was no thrombosis at the infarct site in coronary angiography after treatment in both groups. The efficacy in the observation group and control group were 89.4% and 81.8%, respectively; there was no significant difference between the two groups. The incidence of re-thrombosis in the two groups was 1.5% and 12.1% respectively, which was significantly lower in the observation group than in the control group (P<0.05). The cardiac function indicator of both groups improved after treatment, especially the observation group (P<0.05). There was no significant difference in prothrombin time (PT), activated partial thromboplastin time (APTT), prothrombin activity (PA) and platelet aggregation rate (PAR) in the two groups before treatment (P>0.05). There was also no significant difference in PT and PA before and after treatment (P>0.05). The APTT and PAR were significantly different after treatment (P<0.05), and the PAR of the observation group was significantly higher than that of the control group (P<0.05). The incidence of adverse reactions in the observation group was 7.58%, which was not significantly different with that of the control group (12.12%) (P<0.05).

**Conclusion:**

Aspirin combined with clopidogrel can effectively reduce the occurrence of re-thrombosis after PCI and improve the recovery of cardiac function after acute operation, moreover the safety is high. It has important clinical application values.

## INTRODUCTION

Acute myocardial infarction is defined as partial acute necrosis of the myocardium caused by persistent and severe myocardial ischemia. It is characterized by severe and persistent retrosternal pain, increased serum myocardial enzymes activity and progressive electrocardiographic changes. Some patients may also suffer from arrhythmia, shock and heart failure, which seriously threatens the lives of patients.^1^,^2^ With the aggravation of aging, the improvement of people’s living standard and the change of life style, the incidence of coronary atherosclerotic heart disease is increasing year by year, and the age of patients becomes younger.^3^,^4^ At present, the most effective treatment for acute myocardial infarction is percutaneous coronary intervention (PCI), which can effectively relieve the clinical symptoms of patients.^5^,^6^ However, a large number of studies have shown that patients with acute myocardial infarction are prone to re-thrombosis or vascular stenosis after receiving PCI.^7^,^8^ Therefore, post-PCI continuous antithrombotic therapy is very important. Currently aspirin is commonly used in clinic, but it is limited in clinical application because of its slow onset and high variability.^9^ In this study, 132 patients with acute myocardial infarction were taken as research subjects to study the clinical efficacy of aspirin combined with clopidogrel after PCI.

## METHODS

One hundred and thirty-two patients with acute myocardial infarction who were admitted to our hospital from December 2016 to December 2017 were selected as subjects. Inclusive criteria included satisfying the diagnostic criteria of acute myocardial infarction,^10^ the duration of myocardial infarction not more than 12 hours, having PCI indication and cardiac function Killip grade I-II. Exclusive criteria included having cardiomyopathy, pericarditis, aortic dissection or acute pulmonary embolism, having severe heart failure and hemodynamic instability, having active hemorrhage, coagulation dysfunction and malignant tumor and having cognitive and language dysfunction.

The patients were divided into two groups according to the random number table method, 66 each group. In the observation group there were 42 males and 24 females, aged 45-81 years (average (62.45±11.39) years). The interval between onset of symptoms and outpatient was 1-12 hours (average (3.17±1.46) hours). As to the grade of cardiac function, there were 29 cases of grade I and 37 cases of grade II. The control group consisted of 39 males and 27 females. They aged 46-81 years (average (62.72±12.26) years); the interval between onset of symptoms and chest pain was 1-12 hours (average (3.24±1.37) hours). As regards the grade of cardiac function, there were 26 cases of grade I and 40 cases of grade II. The difference of clinical baseline data between the two groups was not statistically significant. The study was approved by the ethics committee of our hospital and the informed consent was signed by all the selected patients.

Patients in both groups underwent PCI under the guidance of coronary angiography and received conventional drug therapy associated with PCI. Continuous nursing intervention was carried out after operation. Firstly, a continuous intervention group which was composed of attending doctors, competent nurses and head nurses was set up, and the group members were trained comprehensively, including communication skills, knowledge of continuous nursing and specialty knowledge about PCI for acute myocardial infarction. The disease file of the patients including name, sex, nationality, education level, address, contact information and drug use after discharge was established. Family visit was carried out once a month, and telephone follow-up was done once a week. The patients were guided to eat low-salt and low-fat diet and fresh vegetables and fruits and give up smoking and alcohol, to take exercise progressively, take antiplatelet, statin and antihypertensive and hypoglycemic drugs. The existence of adverse drug reactions were evaluated and interfered. The communication with patients and their families was strengthened. Effective psychological intervention was carried out to alleviate the anxiety and depression of patients. A lecture was held once a month and the chat groups were established to inform patients with disease-related knowledge including the knowledge of acute myocardial infarction, the significance of drug treatment, common adverse drug reactions, treatment, observation and emergency treatment of angina pectoris.

The patients in the control group were given 300 mg of aspirin enteric-coated tablets immediately after admission (national approval number: J20130078; Bayer Health Care Pharmaceutical Co., Ltd., China; specification: 100 mg/tablet; production batch no.: BJ18164, BJ19219, BJ25122). Emergency PCI treatment was performed. The patient was intravenously injected with ordinary heparin for anticoagulation during the operation. After the operation, the patient was subcutaneously injected with 5000 U of low molecular weight heparin sodium, once every 12 hours, for 5-7 days, and intravenously infused with 15 μg/(kg·min) tirofiban hydrochloride, for 24 h. Moreover the patient also orally took aspirin, 100 mg each time, once a day. The treatment lasted for 4 months.

Patients in the observation group were given 600 mg of clopidogrel bisulfate tablets immediately after admission (national approval number: J20130083; Sanofi-Aventis (Hangzhou) Pharmaceutical Co., Ltd., China; specification: 75 mg/tablet; batch number: 3A579, 4A757, 5A458) and 300 mg of aspirin enteric-coated tablets. Emergency PCI was performed.

During the operation, the patient was intravenously injected with ordinary heparin for anticoagulation. After operation, the patient was subcutaneously injected with 5000 U of low molecular weight heparin sodium, once every 12 hours, for 5-7 days, and intravenously infused with 15 μg/(kg·min) tirofiban hydrochloride, for 24 hour similar to the control group. Moreover the patient also orally took 75 mg of clopidogrel bisulfate tablets and 100 mg of aspirin enteric-coated tablets daily. The treatment lasted for four months.

### Observational Indicators

The results of electrocardiography and coronary angiography were observed and recorded, and the clinical effects of the two groups were evaluated. The criteria of curative effect were as follows. Treatment was assessed as significantly effective if ST segment dropped for more than 50% within one hour after operation and recovered to the baseline level within seven days after operation, as effective if ST segment dropped for less than 50% but more than 20% within two hours after operation and recovered to the baseline level within 14 days after operation, and as ineffective if ST segment dropped less than 20% within 2 h after operation. The total effectiveness could be calculated using the formula: the total effective rate=(significantly effective number+effective number)/the total number of cases. The occurrence of re-thrombosis in the two groups was evaluated using coronary angiography. The improvement of cardiac function in the two groups was observed before and after treatment, and the relevant cardiac function indicators included left ventricular end-diastolic diameter (LVEDD) and left ventricular end-systolic diameter (LVESD). Coagulation indicators including partial prothrombin time (PT), activated partial thrombin time (APTT) and prothrombin activity (PA) were measured using freezing method. Platelet aggregation rate (PAR) was measured by turbidimetry. The incidence of adverse reactions was compared between the two groups.

The data were processed by SPSS 21.0. The measurement data were expressed by Mean±SD and processed by t test. The counting data were expressed by number of cases (percentage) processed by and X^2^ test. P<0.05 suggested that the difference was statistically significant.

## RESULTS

There was no thrombosis at the infarct site after coronary angiography in both groups. The total effective rate of the observation group and the control group was 89.4% and 81.8%, respectively with no significant difference. As regards re-thrombosis the observation group was significantly lower than that in the control group (1.5% vs. 12.1%) (P<0.05, Table-I).

**Table-I:** Clinical efficacy and incidence of re-thrombosis [n(%)].

Group	Observation group	Control group	X^2^	P
Significantly effective	39(59.1)	35(53.0)		
Effective	20(30.3)	19(29.8)		
Ineffective	7(10.6)	12(18.2)		
Overall effective rate	59(89.4)	54(81.8)	0.0216	>0.05
Incidence of re-thrombosis	1(1.5)	8(12.1)	9.9731	<0.05

Before treatment, there was no significant difference in LVEDD and LVESD between the two groups (P>0.05). After treatment, the decrease of LVEDD and LVESD in the observation group was more significant than that in the control group, and the difference was statistically significant (P<0.05, Table-II).

**Table-II:** Cardiac function indicators between the two groups before and after treatment.

Group		Observation group	Control group
LVEDD	Before treatment	69.50±9.86	69.45±9.91
	After treatment	47.00±7.51^*#^	68.01±8.49
LVESD	Before treatment	58.00±10.51	57.96±10.54
	After treatment	36.05±7.50^*#^	56.00±10.55

***Note:***

* indicated P<0.05 compared to before treatment;

# indicated P<0.05 compared to the control group.

There was no significant difference in PT, a PTT, PA and PAR between the two groups before treatment (P>0.05). There was no significant difference in PT and PA after treatment (P>0.05). There was significant difference in APTT and PAR between the two groups before treatment (P<0.05). The PAR of the observation group was significantly higher than that of the control group (P<0.05, Table-III).

**Table-III:** Coagulation indicators and platelet aggregation rate.

Group		Observation group	Control group
PT	Before treatment	11.32±2.68	11.24±2.57
	After treatment	12.01±3.38	11.59±3.41
APTT	Before treatment	33.61±5.63	33.52±5.87
	After treatment	39.44±6.57^*^	39.21±6.18^*^
PA	Before treatment	0.88±0.06	0.89±0.07
	After treatment	0.89±0.07	0.89±0.08
PAR	Before treatment	0.58±0.08	0.59±0.07
	After treatment	0.46±0.08^*#^	0.33±0.06^*#^

***Note:***

* indicated P<0.05 compared to before treatment;

# indicated P<0.05 compared to the control group.

During the treatment, five patients had adverse reactions in the control group, including two cases of nausea, one case of epigastric discomfort, 1 case of rash and 1 case of headache (7.58%, 5/66), and 8 patients had adverse reactions in the observation group, including 3 cases of nausea, 1 case of headache, 2 cases of abdominal pain and 2 cases of rash (12.12%, 8/66). There was no significant difference in the incidence of adverse reactions between the two groups (X^2^=0.447, P<0.05).

## DISCUSSION

The pathological background of myocardial infarction is rupture of unstable plaque of coronary artery or secondary thrombosis. Therefore clinical acute symptoms such as acute myocardial infarction may happen when thrombogenesis progresses to a certain degree. The main principle of clinical treatment of acute myocardial infarction is to open the related infarct vessels as soon as possible to promote the recovery of myocardial blood flow and rescue the dying myocardium.^11^,^12^ Emergency PCI is one of the effective measures for the treatment of acute myocardial infarction.^13^ In emergency PCI, trauma is mild, the opening rate of infarct vessels is more than 90%, and there is no absolute contraindication.^14^ But in the process of PCI, balloon dilatation, stent implantation and other instruments are needed, which can exert pressure on the wall of coronary artery and then induce plaque rupture and intima and media injury of coronary artery. Intima and media injury of coronary artery can activate platelets and promote platelet adhesion and aggregation to form thrombus.^15^,^16^ Therefore, hemolysis and antithrombotic therapy are the keys to successful PCI. In the past, aspirin was used more frequently. The antiplatelet mechanism of aspirin is to make arachidonic acid lose its ability to transform into prostaglandin endoperoxide and hindering the formation of prostaglandin E2 and thromboxane A2 through inhibiting the activity of cyclooxygenase in platelets.^17^ However, a study shows that aspirin resistance exists in a few patients although aspirin has a good therapeutic effect in the treatment of acute myocardial infarction,^18^ that is, acute thrombosis may occur even after taking aspirin, and the incidence of adverse reactions increases with the increase of dosage. Therefore, aspirin cannot reduce acute thrombotic events in clinical application.

Clopidogrel is a highly effective antiplatelet drug. It is a new type of thiophene pyridine preparation. Its antiplatelet mechanism is different from the action of aspirin on prostaglandin E2 and thromboxane A2. Clopidogrel inhibits the combination of adenosine diphosphate-mediated blood platelet glycoprotein II b/III and fibrinogen receptor irreversibly by binding with adenosine diphosphate, thereby inhibiting blood platelet aggregation and reducing thrombus.^19^,^20^ A study shows that aspirin will not affect the inhibition of adenosine diphosphate-induced platelet aggregation when combining with clopidogrel,^21^ clopidogrel can strengthen the inhibitory effect of aspirin on prostaglandin E2 and thromboxane A2 pathways, and the combination of clopidogrel and aspirin can inhibit blood platelet aggregation more effectively.

The results of this study showed that there was no thrombosis at the infarct site after coronary angiography in the two groups; there was no significant difference in the total effective rate between the two groups; the incidence of re-thrombosis in the observation group was significantly lower than that in the control group after treatment, indicating that aspirin combined with clopidogrel could effectively reduce the incidence of re-thrombosis after PCI. Moreover, the improvement of cardiac function index in the observation group was significantly better than that in the control group, which was consistent with the results of Xiao et al.^22^ It showed that the combination of drugs could significantly improve the recovery of cardiac function in patients with acute myocardial infarction. In addition, there were no significant changes in PT and PA in the observation group after treatment, APTT increased and PAR decreased compared to before treatment, and APPT and PAR of the observation group was significantly higher than that in the control group. It suggested that aspirin combined with clopidogrel could effectively reduce aspirin resistance and improve myocardial perfusion, with high safety, and the long-term efficacy of aspirin combined with clopidogrel might be better than that of clopidogrel alone.

## CONCLUSION

Considering the characteristics of acute myocardial infarction, the treatment based on aspirin combined with clopidogrel has better clinical efficacy than aspirin alone. The therapy can effectively improve the cardiac function of patients. Moreover the incidences of complications and adverse events were low, and the treatment safety was high. The sample size of this study was small, and larger sample size is needed in further studies.

### Authors’ Contribution

**XYZ & YXL:** Study design, data collection and analysis.

**XYZ, LZQ & YXL:** Manuscript preparation, drafting and revising.

**XYZ:** Review and final approval of manuscript.
